# Supplementary material for: Combinatorial GxGxE CRISPR screen identifies SLC25A39 in mitochondrial glutathione transport linking iron homeostasis to OXPHOS
Source: Nat Commun. 2022 May 5;13:2483. doi: 10.1038/s41467-022-30126-9 (PMC9072411; doi:10.1038/s41467-022-30126-9)
Supplement: Supplementary file 1 — Supplementary Information [file 41467_2022_30126_MOESM1_ESM.pdf]

# Combinatorial *GxGxE* CRISPR screen identifies SLC25A39 in mitochondrial glutathione transport linking iron homeostasis to OXPHOS

**Xiaojian Shi[1,5], Bryn Reinstadler[2,3,4,5], Hardik Shah[2,3,4], Tsz-Leung To[2,3,4], Katie Byrne[1], Luanna Summer[1], Sarah E. Calvo[2,3,4], Olga Goldberger[2,3], John G. Doench[4], Vamsi K. Mootha[2,3,4], Hongying Shen[1*]**

[1] Cellular and Molecular Physiology Department, Yale School of Medicine, New Haven, CT 06511, USA; Systems Biology Institute, Yale West Campus, West Haven, CT 06516, USA
[2] Howard Hughes Medical Institute and Department of Molecular Biology, Massachusetts General Hospital, Boston, MA 02114, USA
[3] Department of Systems Biology, Harvard Medical School, Boston, MA 02115, USA
[4] Broad Institute, Cambridge, MA 02141, USA
[5] These authors contributed equally to this work

[*] Correspondence: hongying.shen@yale.edu (H.S.)

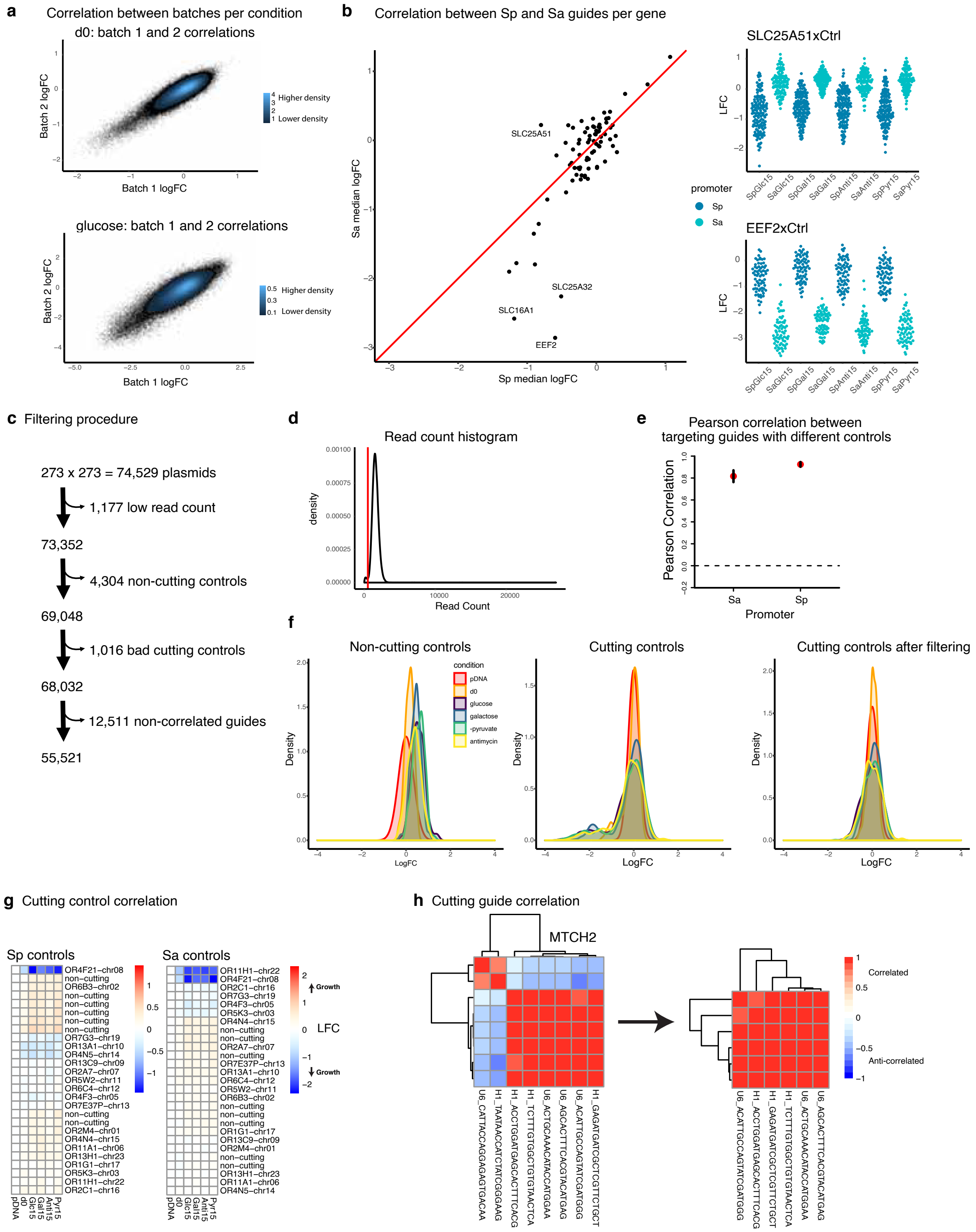


**Supplementary Fig. 1 Data analysis for the combinatorial CRISPR screen of SLC25 family.** **a**, The correlations between the LFC values of two screen batches on day 0 and day 15 in glucose with the superimposed contour plot to show point density. Lighter blue means higher density of

points. **b,** (Left) The correlation of LFC values of the average single gene KO fitness between the Ctrl x Gene vs Gene x Ctrl. Labels indicate a very small number of genes that fall off the diagonal. (Right) For instance, SLC25A51 has a no-growth phenotype when knocked out by targeting guides at SaCas9 position, and a growth inhibition phenotype when knocked out by targeting guides at SpCas9 position. EEF2 shows a more extreme deleterious phenotype when knocked out by SaCas9 guides compared to SpCas9 guides. **c,** The filtering procedure for data analysis (see details in the methods). **d,** Read count histogram for all guide pairs in the plasmid DNA; an empirical cut-off at 500 reads is indicated with a red line. **e,** The Pearson correlation of the single gene KO fitness phenotype between the targeting 17 SpCas9 guides (Gene x Ctrl) (n=289) vs the 15 SaCas9 guides (Ctrl x Gene) (n=225). **f,** The density plots for the LFC value of non-cutting controls (left), cutting controls (middle), and cutting controls after filtering (right). Different conditions are color-coded (pDNA, red; day 0, orange; glucose, purple; galactose, blue; -pyruvate, green; antimycin, yellow). **g,** Heatmaps showing the LFC of the cutting negative control guides across the different conditions. Cutting control guides with deleterious fitness phenotype were filtered from both SaCas9 and SpCas9 positions. **h,** The Pearson correlation of SpCas9 and SaCas9 guides targeting the same single gene (either Gene x Ctrl or Ctrl x Gene) across different conditions were calculated. Guides that showed anti-correlation were filtered out for subsequent analysis. Source data are provided.

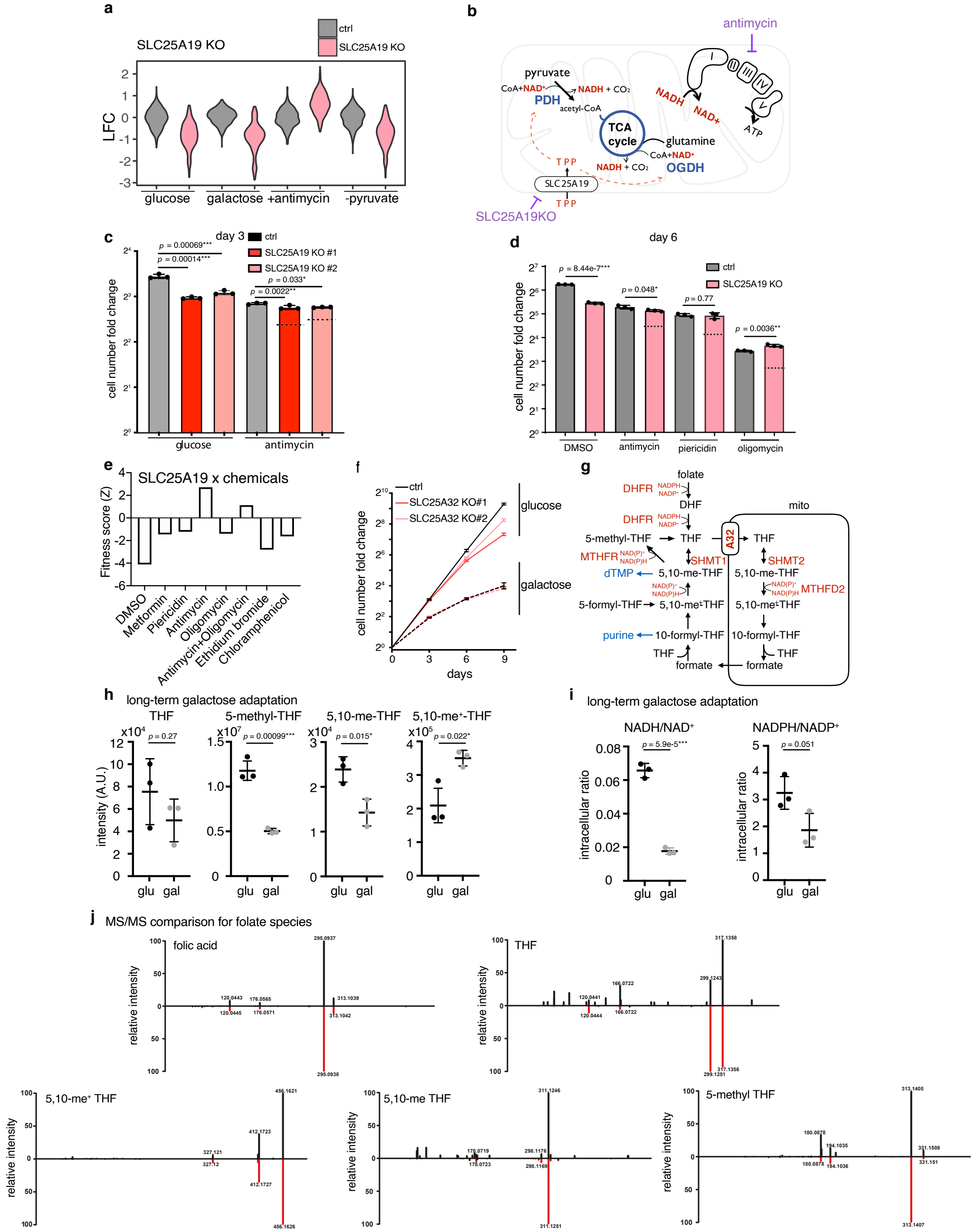


**Supplementary Fig. 2 Single KO gene by environment interaction for the two hits, SLC25A19 and SLC25A32. a,** The growth fitness of SLC25A19 single CRISPR KO cells and controls in the screen. Grey is the Log Fold Change (LFC) of the Ctrl x Ctrl cells, and pink is LFC of the SLC25A19 KO (SLC25A19 x Ctrl or Ctrl x SLC25A19). **b,** Diagram depicting mitochondrial ThPP metabolism. SLC25A19 is the mitochondrial carrier for ThPP, the cofactor

critical for matrix PDH and OGDH complexes that generate reduced NADH for the ETC. **c-d,** Follow-up study confirmed the growth defect of SLC25A19 CRISPR KO cells using two different sgRNA guides (in c), and the defect is buffered in the antimycin, piericidin, and oligomycin conditions (in d) (n=3). **e**, reanalysis of SLC25A19 result from the ETC inhibitor-dependent genome-wide CRISPR screen. **f,** The growth curve of SLC25A32 KO and control cells in glucose ($p_{ctrl/A32KO\#1}$=7.74×10$^{-6}$, $p_{ctrl/A32KO\#2}$=5.46×10$^{-5}$, day=9) and galactose ($p_{ctrl/A32KO\#1}$=0.66, $p_{ctrl/A32KO\#2}$=0.18, day=9) (n=3). **g**, cellular folate metabolic pathway. **h**, Long-term adaptation in galactose for 2 weeks reduced most tetrahydrofolate species, with the exception of 5,10-me$^{+}$-THF (n=3). **i**, Long-term adaptation in galactose for 2 weeks reduced the cellular NADH/NAD$^{+}$ and NADPH/NADP$^{+}$ ratio (n=3). **j**, MS/MS comparison of folate species detected in the samples (black) and standards (red). Statistical significance was calculated using two-tailed *t*-test. Significance level were indicated as *** $p < 0.001$, ** $p < 0.01$, * $p < 0.05$ and n.s. $p > 0.05$. Data are expressed as mean ± SD. Source data are provided.

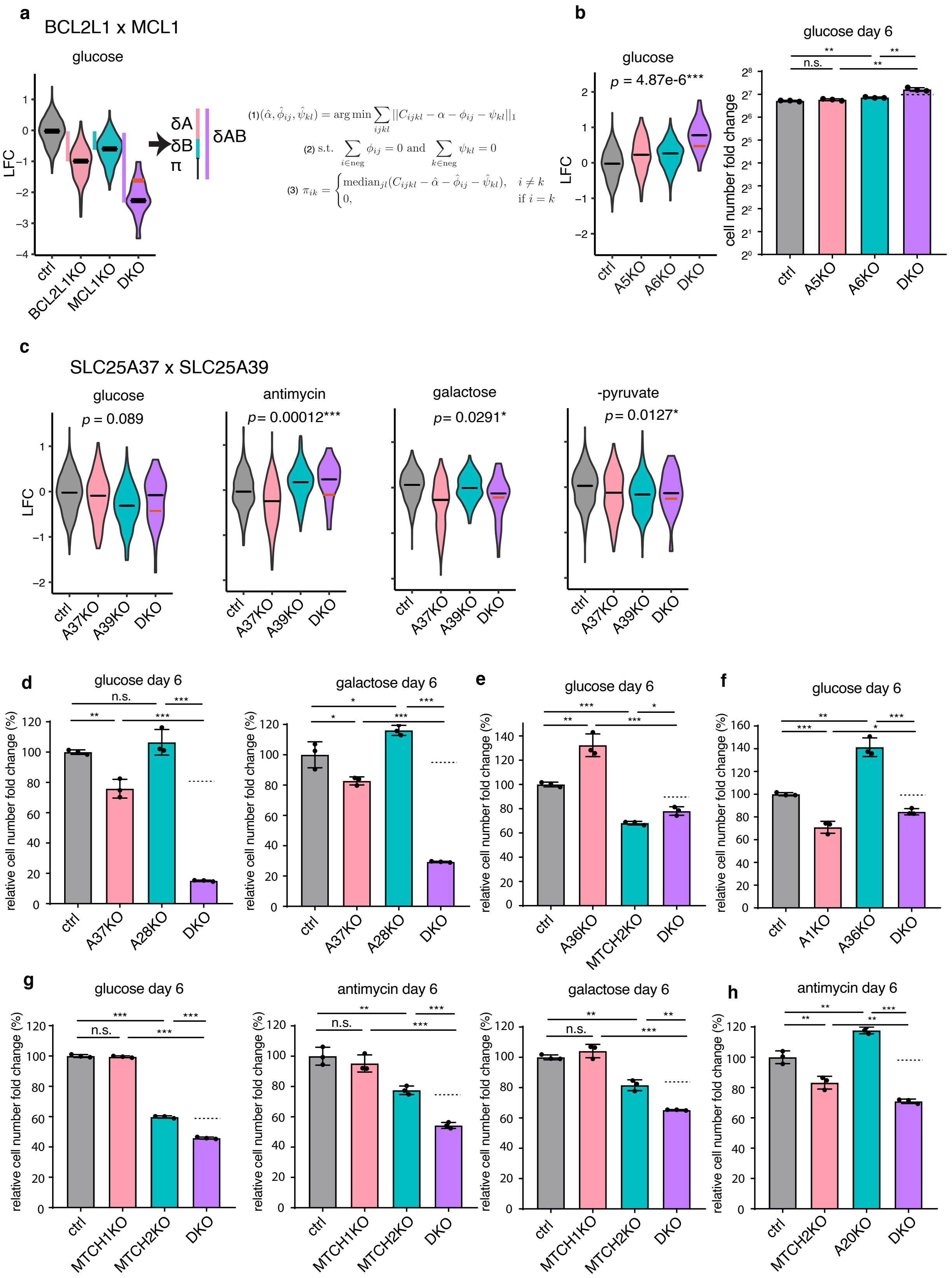

a
BCL2L1 x MCL1
glucose
LFC
ctrl
BCL2L1KO
MCL1KO
DKO
δA
δB
π
δAB
(1) $(\hat{\alpha}, \hat{\phi}_{ij}, \hat{\psi}_{kl}) = \arg\min \sum_{ijkl} ||C_{ijkl} - \alpha - \phi_{ij} - \psi_{kl}||_1$
(2) s.t. $\sum_{i \in neg} \phi_{ij} = 0$ and $\sum_{k \in neg} \psi_{kl} = 0$
(3) $\pi_{ik} = \begin{cases} \text{median}_{jl}(C_{ijkl} - \hat{\alpha} - \hat{\phi}_{ij} - \hat{\psi}_{kl}), & i \neq k \\ 0, & \text{if } i = k \end{cases}$
b
glucose
p = 4.87e-6***
A5KO
A6KO
glucose day 6
cell number fold change
n.s.
c
SLC25A37 x SLC25A39
glucose
p = 0.089
antimycin
p= 0.00012***
galactose
p= 0.0291*
-pyruvate
p= 0.0127*
A37KO
A39KO
d
glucose day 6
galactose day 6
relative cell number fold change (%)
A28KO
e
A36KO
MTCH2KO
f
A1KO
g
antimycin day 6
MTCH1KO
h
A20KO

**Supplementary Fig. 3 Validation of Gene x Gene x Environment (GxGxE) interactions.**
**a,** The π-score as a measure of genetic interaction from the screen as reported in Fischer et al. 2015. The π-score is a robust estimation of the difference between the addition of two single knockout phenotypes and the observed double knockout phenotype. The π-score is calculated using the above robust linear model, where, for gene i with guide j, and gene k with guide l, the log fold change of the double knock outs ($C_{ijkl}$) are the data in the matrix. α is an intercept term, and φ and ψ represent the single gene terms. The π-score for a double knock-out of gene i with gene k is the median over all guides j and l of the difference between the log fold change and the estimated parameters. π-scores are defined as 0 for auto-double-knockouts. The positive control pair BCL2L and MCL1 was used to illustrate the calculation. **b,** LFC values from the CRISPR screen are shown to illustrate that SLC25A5 and SLC25A6 double KO cells do not exhibit fitness defect in the glucose, both in the screen (left) and in the follow-up experiments (right) ($p_{ctrl/A5KO}$=0.16, $p_{ctrl/A6KO}$=0.0027, $p_{A5KO/DKO}$=0.0013, $p_{A6KO/DKO}$=0.0025) (n=3). **c,** LFC values from the screen are shown to illustrate the interaction between SLC25A37 and SLC25A39 loss in the glucose and antimycin conditions. The red line indicates the expected LFC, based on an additive model, if there were no genetic interaction. The black line indicates the actual median LFC value. **d-h,** follow-up experimental validation of the GxG hits from the CRISPR screen (n=3). **d,** glucose condition ($p_{ctrl/A37KO}$ =0.0027, $p_{ctrl/A28KO}$=0.26, $p_{A37KO/DKO}$=$7.02\times10^{-5}$, $p_{A28KO/DKO}$=$4.81\times10^{-5}$), galactose condition ($p_{ctrl/A37KO}$=0.029, $p_{ctrl/A28KO}$=0.039, $p_{A37KO/DKO}$=$4.2\times10^{-6}$, $p_{A28KO/DKO}$=$1.48\times10^{-6}$). **e,** ($p_{ctrl/A36KO}$ =0.0042, $p_{ctrl/MTCH2KO}$ =$2.17\times10^{-5}$, $p_{A36KO/DKO}$ =0.00071, $p_{MTCH2KO/DKO}$=0.011). **f,** ($p_{ctrl/A1KO}$ =0.00074, $p_{ctrl/A36KO}$=0.0010, $p_{A1KO/DKO}$=0.016, $p_{A36KO/DKO}$=0.00035). **g,** glucose condition ($p_{ctrl/MTCH1KO}$=0.48, $p_{ctrl/MTCH2KO}$=$5.84\times10^{-7}$, $p_{MTCH1KO/DKO}$=$4.97\times10^{-8}$, $p_{MTCH2KO/DKO}$=$1.96\times10^{-5}$), antimycin condition ($p_{ctrl/MTCH1KO}$=0.37, $p_{ctrl/MTCH2KO}$=0.0040, $p_{MTCH1KO/DKO}$=0.0003, $p_{MTCH2KO/DKO}$=0.00031), galactose condition ($p_{ctrl/MTCH1KO}$=0.20, $p_{ctrl/MTCH2KO}$=0.0012, $p_{MTCH1KO/DKO}$=0.00011, $p_{MTCH2KO/DKO}$=0.0014). **h,** ($p_{ctrl/MTCH2KO}$=0.0081, $p_{ctrl/A20KO}$=0.0029, $p_{MTCH2KO/DKO}$=0.0089, $p_{A20KO/DKO}$=$6.7\times10^{-6}$). The dotted line indicates the expected growth fitness, based on an additive model, if there were no specific genetic interaction. Statistical significance was calculated using two-tailed *t*-test. Significance level were indicated as *** $p < 0.001$, ** $p < 0.01$, * $p < 0.05$ and n.s. $p > 0.05$. Data are expressed as mean ± SD. Source data are provided.

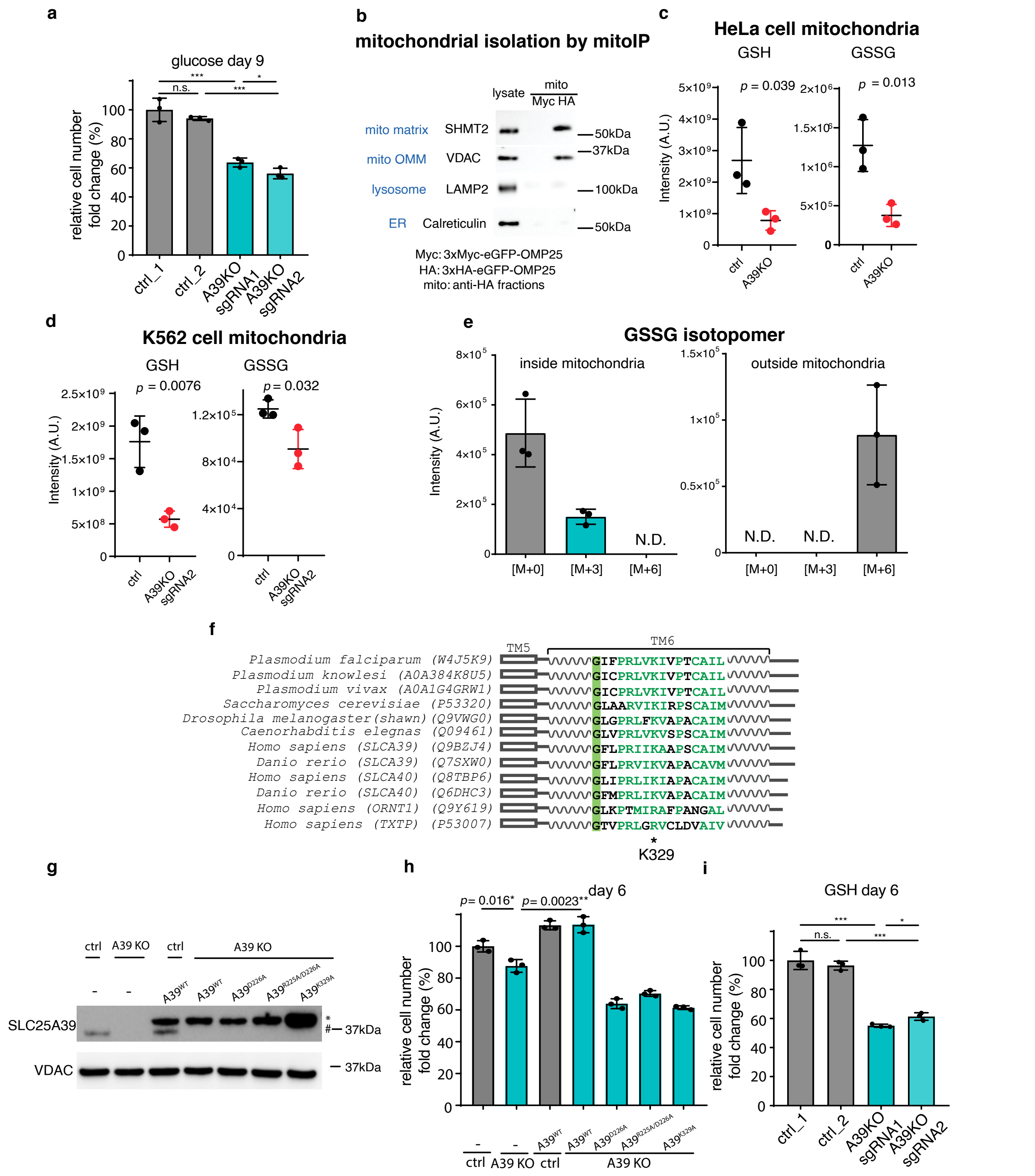


**Supplementary Fig. 4 Validation of growth fitness defect involving SLC25A39 and functional follow-up. a,** Growth fitness of SLC25A39 CRISPR KO cells generated by two different sgRNAs cultured in glucose condition ($p_{\text{ctrl-1/ctrl-2}}$=0.27, $p_{\text{ctrl-1/sgRNA1}}$=0.0018, $p_{\text{ctrl-2/sgRNA2}}$=$7.17\times10^{-5}$, $p_{\text{sgRNA1/sgRNA2}}$=0.052) (n=3). **b,** Western blotting of organelle markers, SHMT2 (matrix), VDAC (outer-mitochondrial membrane, OMM), LAMP2 (lysosome) and Calreticulin (endoplasmic

reticulum, ER), from the whole cell lysates and anti-HA immuno-isolated fractions from the cells expressing 3xMyc-eGFP-OMP25 (Myc) and 3xHA-eGFP-OMP25 (HA), confirming selective enrichment for the mitochondria. **c,** LC-MS measurement of mitochondrial GSH and GSSG levels in the control and A39 KO HeLa cell mitochondria (n=3). **d,** LC-MS measurement of mitochondrial GSH and GSSG levels in the control and A39 KO K562 cells, in which a different A39-targeting sgRNA guide was used (n=3). **e,** GSSG isotopomer distribution from the inside (washed mitochondria fraction) and the outside (assay supernatant) of the A39 KO mitochondria in the GSH uptake assay at the 30 min time point (n=3). **f,** Multiple sequence alignment of the eukaryotic A39 ortholog sequences spanning diverse taxa, as well as the human amino acid ornithine transporter SLC25A15 (ORNT1) and the human citrate transporter SLC25A1 (TXTP). The position for the human SLC25A39 Lys329, a positive residue previously shown to be responsible for mediating solute-induced conformational changes and critical for transport activity is labeled with an asterisk. **g,** Western blotting showing the endogenous A39 (#) and ectopically expressed A39 protein (*). VDAC was used as the loading control. **h,** Rescue of growth fitness defect in the A39 KO cells by wild type $A39^{WT}$ versus A39 mutants predicted to be defective either in GSH binding ($A39^{D226A}$, $A39^{R225A/D226A}$) or in conformational changes ($A39^{K329A}$) (n=3). **i,** Growth fitness defect in the SLC25A39 CRISPR KO cells is not rescued by supplementation with 10mg/L GSH ($p_{ctrl\text{-}1/ctrl\text{-}2}$=0.43, $p_{ctrl\text{-}1/sgRNA1}$=0.00025, $p_{ctrl\text{-}2/sgRNA2}$=0.00011, $p_{sgRNA1/sgRNA2}$=0.018) (n=3). Statistical significance was calculated using two-tailed *t*-test. Significance level were indicated as *** $p < 0.001$, ** $p < 0.01$, * $p < 0.05$ and n.s. $p > 0.05$. Data are expressed as mean ± SD. Source data are provided.

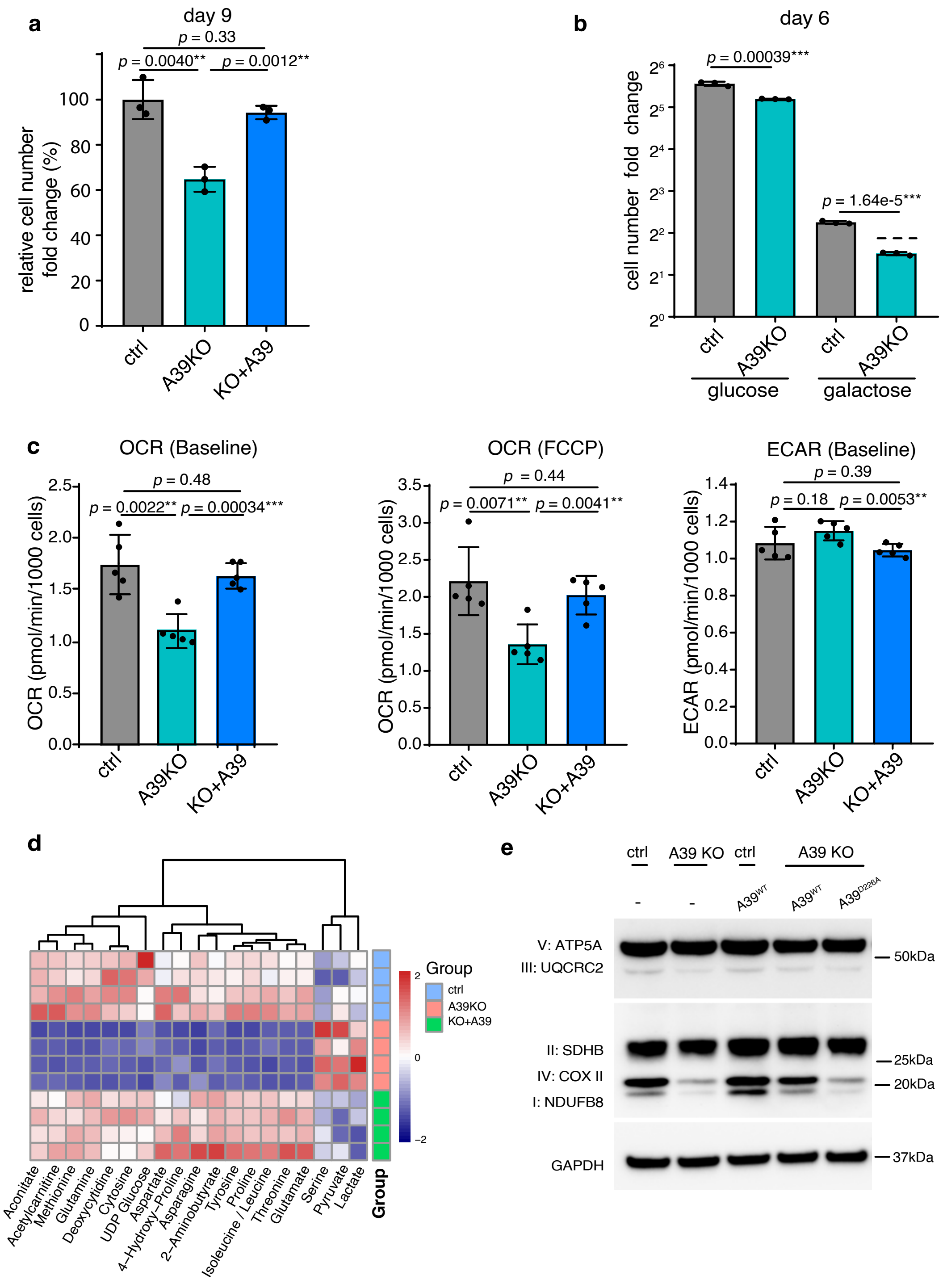


**Supplementary Fig. 5 Mitochondrial glutathione depletion upon SLC25A39 KO leads to defective mitochondrial bioenergetics. a,** Growth fitness defect in the SLC25A39 CRISPR KO cells is rescued by re-expressing CRISPR-resistant SLC25A39 (n=3). **b,** SLC25A39 KO cells exhibited stronger growth fitness defect in galactose condition, supporting its role in OXPHOS (n=3). **c,** Respiration phenotypes (Basal OCR, maximal respiration under FCCP treatment and basal ECAR) in the SLC25A39 KO are rescued by re-expressing SLC25A39 (n=3). **d,** Heatmap

showing changes in cellular metabolites caused by SLC25A39 KO and restored by re-expressing SLC25A39. **e,** Western blot of mitochondrial OXPHOS complex subunits showing that the A39 mutant predicted to be defective in GSH binding ($A39^{D226A}$) failed to rescue the phenotype. Statistical significance was calculated using two-tailed *t*-test. Significance level were indicated as *** $p < 0.001$, ** $p < 0.01$, * $p < 0.05$ and n.s. $p > 0.05$. Data are expressed as mean ± SD. Source data are provided.

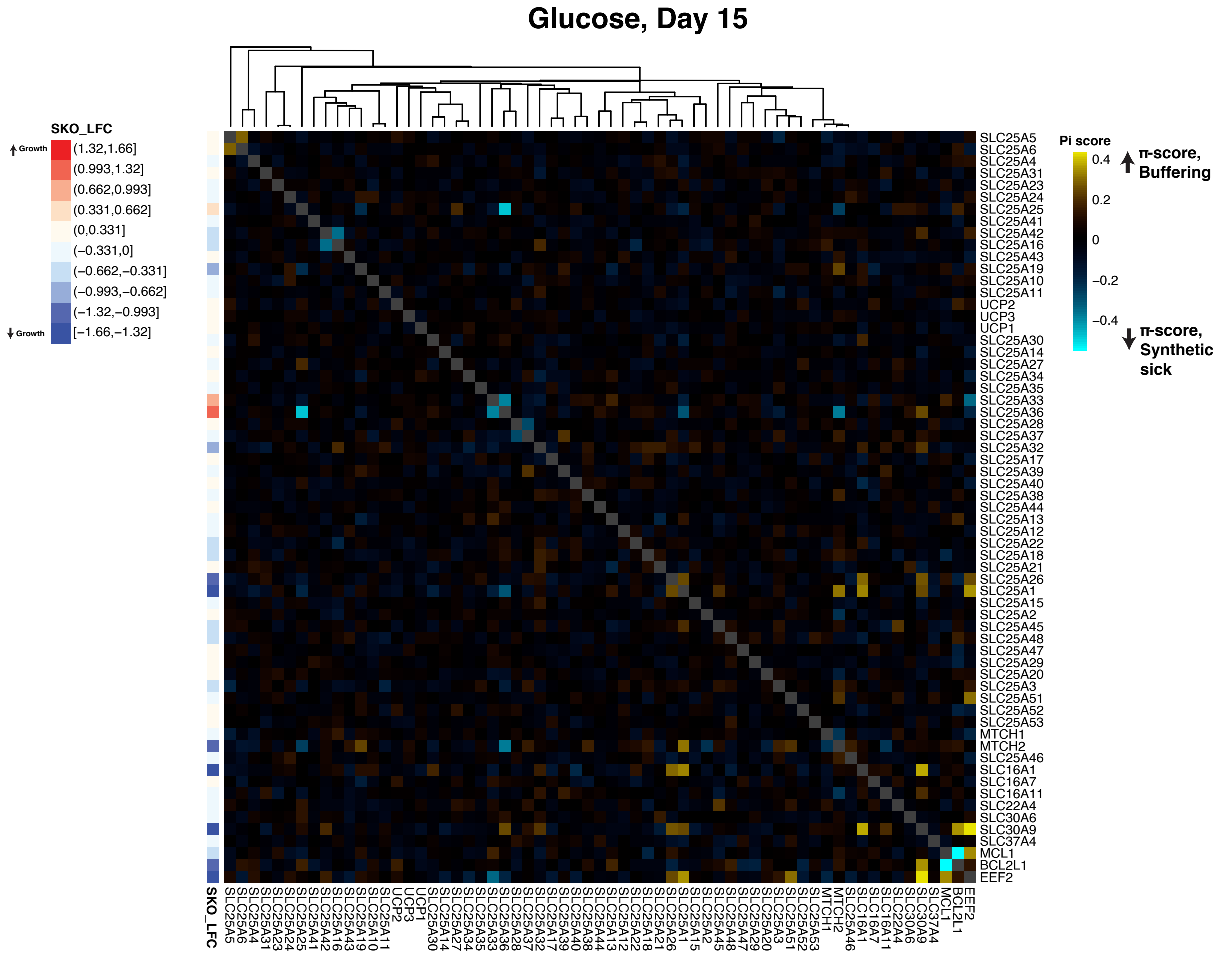


**Supplementary Fig. 6 Genetic interaction for all the gene-pairs in glucose media condition.** Heatmaps with the π-score for each calculated genetic interaction are shown, separated by condition. The single-knockout phenotype of Ctrl x Gene or Gene x Ctrl is annotated on the left side; the top dendrogram indicates the clustering of the SLC25 family members by sequence homology. The diagonal is greyed out. Source data are provided.

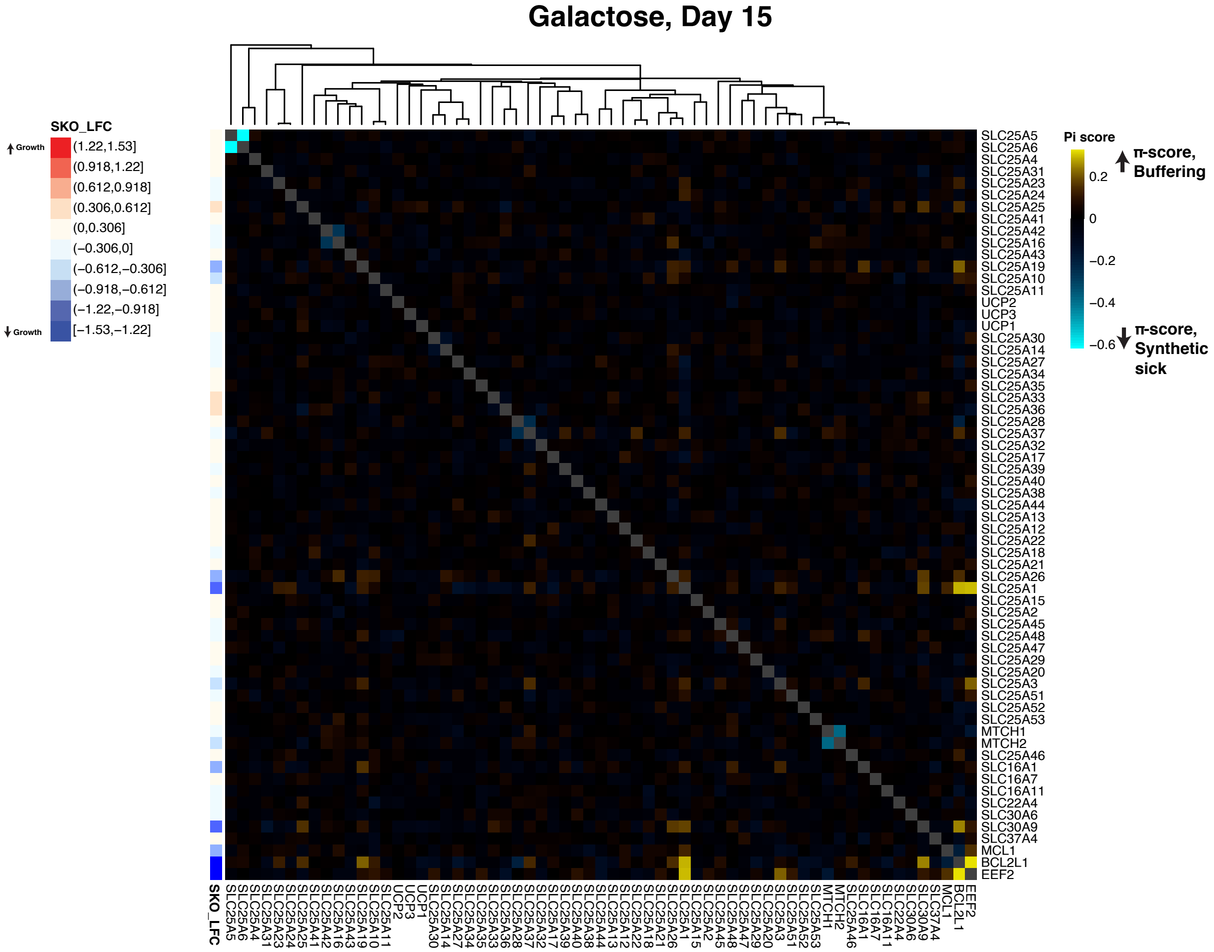


**Supplementary Fig. 7 Genetic interaction for all the gene-pairs in galactose media condition.** Heatmaps with the π-score for each calculated genetic interaction are shown, separated by condition. The single-knockout phenotype of Ctrl x Gene or Gene x Ctrl is annotated on the left side; the top dendrogram indicates the clustering of the SLC25 family members by sequence homology. The diagonal is greyed out. Source data are provided.

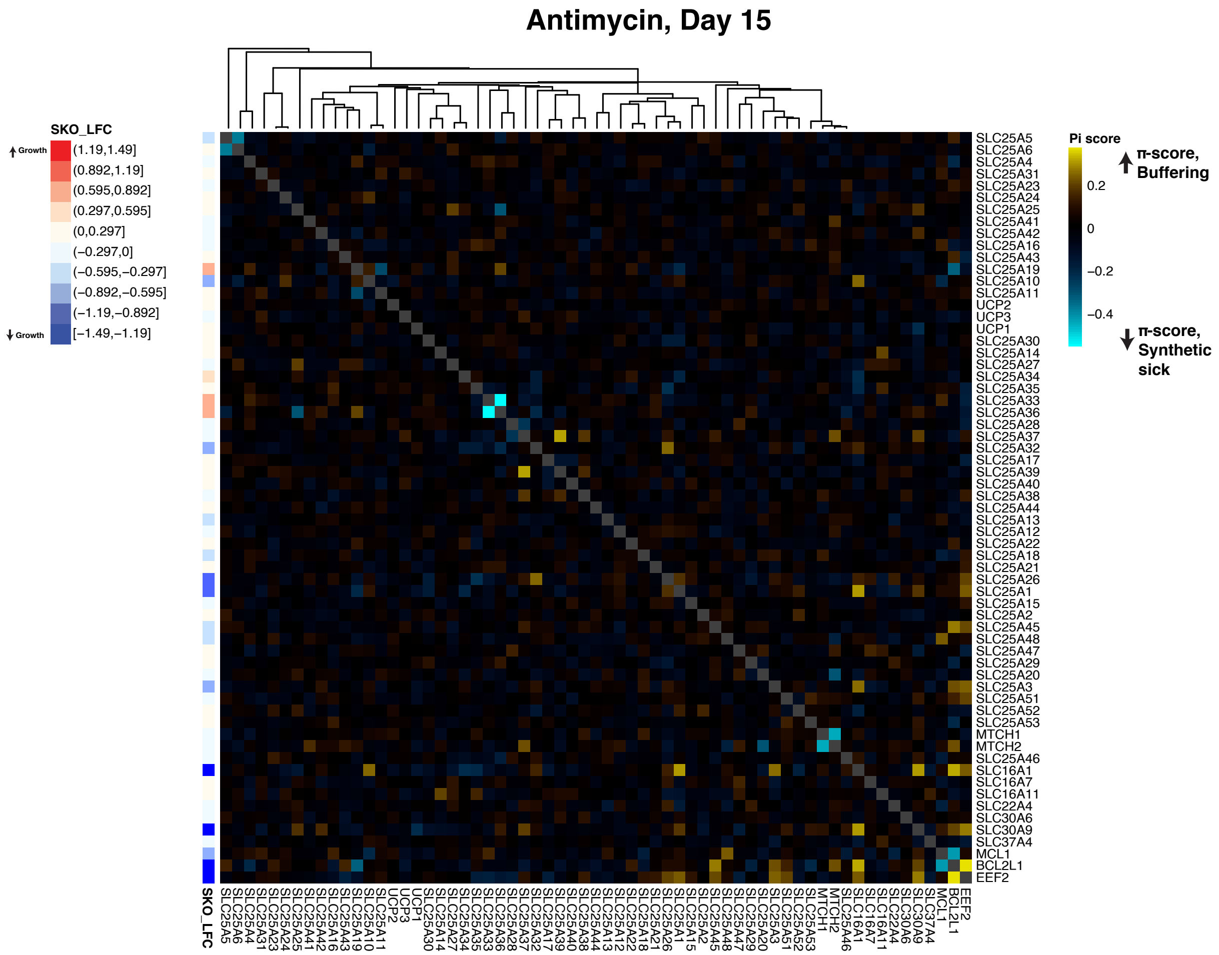


**Supplementary Fig. 8 Genetic interaction for all the gene-pairs in antimycin media condition.** Heatmaps with the π-score for each calculated genetic interaction are shown, separated by condition. The single-knockout phenotype of Ctrl x Gene or Gene x Ctrl is annotated on the left side; the top dendrogram indicates the clustering of the SLC25 family members by sequence homology. The diagonal is greyed out. Source data are provided.

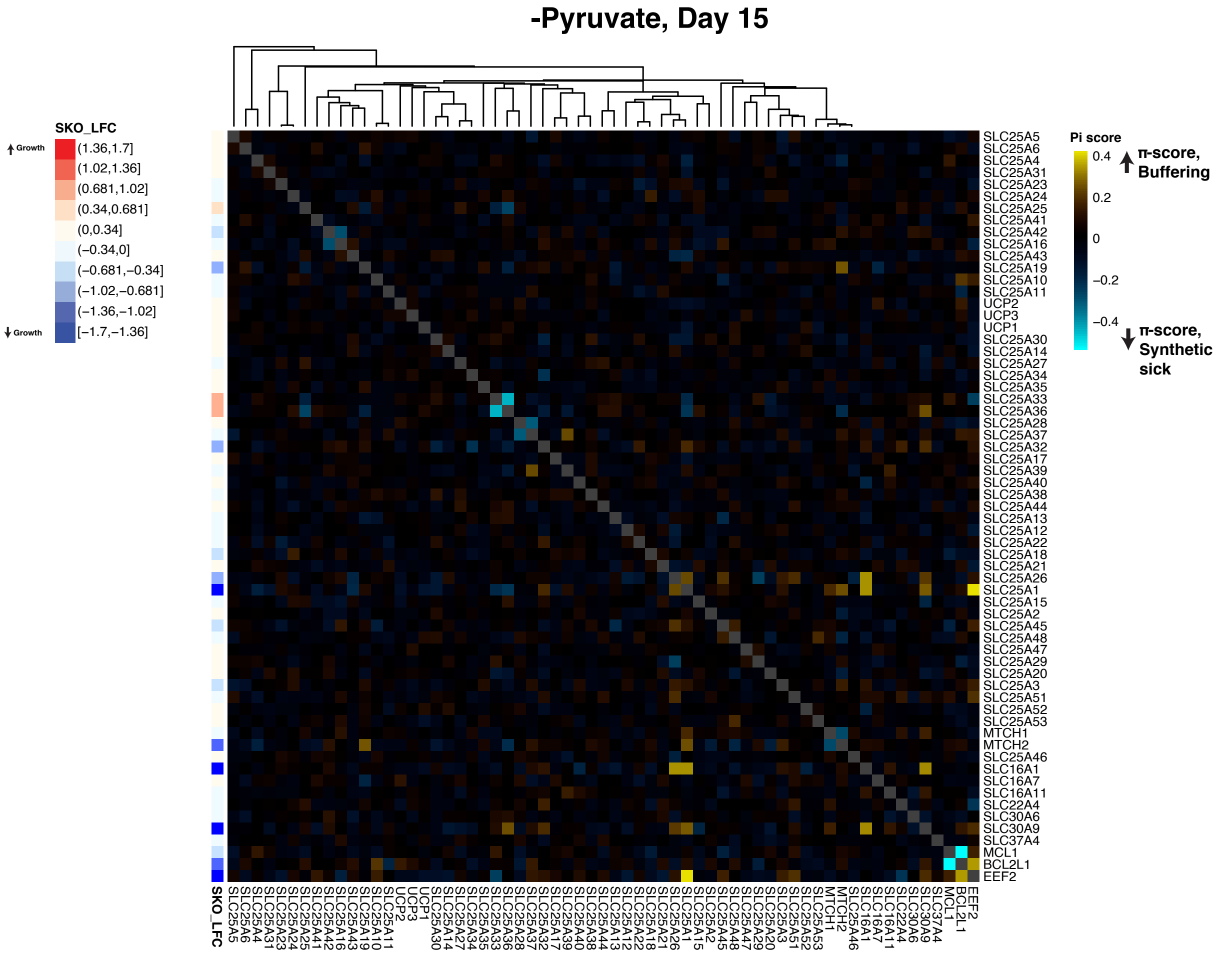


**Supplementary Fig. 9 Genetic interaction for all the gene-pairs in -pyruvate media condition.** Heatmaps with the π-score for each calculated genetic interaction are shown, separated by condition. The single-knockout phenotype of Ctrl x Gene or Gene x Ctrl is annotated on the left side; the top dendrogram indicates the clustering of the SLC25 family members by sequence homology. The diagonal is greyed out. Source data are provided.

**Supplementary Note 1**

**Other observations about the GxGxE screen**

None of the 12 non-expressed SLC25 genes (Supplementary Data 5) were recovered as hits in any of the conditions, supporting the robustness of our CRISPR screen and data analysis pipeline. Our GxE and GxGxE screen hits did not include SLC25 genes that we initially anticipated. For instance, for SLC25A51, a recently identified carrier for NAD cofactor[1-3], our screen confirmed a fitness defect of SLC25A51 KO using the spCAS9 guides (Supplementary Fig. 1b), but did not reveal a GxE interaction in galactose, probably due to redundant mechanisms supporting NAD transport as well as a mild OXPHOS defect of the SLC25A51 KOs in the screen cell line. We did not observe genetic interaction between SLC25A12 and SLC25A13, two Glutamate/Aspartate exchangers in the malate-aspartate shuttle, perhaps because of the functional redundancy with other glutamate carriers SLC25A18 and SLC25A22[4] and the aspartate carrier[5]. Our hits did not include another recently identified branched-chain amino acid (BCAA) carrier SLC25A44[6], probably because BCAA metabolism is dispensable for the K562 cell fitness. In our single gene KO analysis, while the loss of SLC25A25 (ATP-Mg/Pi carrier), SLC25A33 and SLC25A36 (two pyrimidine carriers) conferred a mild growth advantage in the screen (Fig. 2a), we were not able to validate the growth benefit effects of SLC25A33 and SLC25A36 single KOs in the follow-up experiments, presumably due to a "paracrine"-like effect from other genotypes in the pooled screening format. It is unknown if the growth benefit in the SLC25A33 KO in the screen might be linked to a recent proposed role of SLC25A33 in regulating pyrimidine imbalance and innate immunity[7]. In our screen, we did not observe a genetic interaction between SLC25A39 and its paralogous gene SLC25A40.

1. Kory, N. *et al.* MCART1/SLC25A51 is required for mitochondrial NAD transport. *Sci Adv* **6** (2020).
2. Luongo, T. S. *et al.* SLC25A51 is a mammalian mitochondrial NAD(+) transporter. *Nature* **588**, 174-179 (2020).
3. Girardi, E. *et al.* Epistasis-driven identification of SLC25A51 as a regulator of human mitochondrial NAD import. *Nat Commun* **11**, 6145 (2020).
4. Fiermonte, G. *et al.* Identification of the mitochondrial glutamate transporter. Bacterial expression, reconstitution, functional characterization, and tissue distribution of two human isoforms. *J Biol Chem* **277**, 19289-19294 (2002).
5. Vozza, A. *et al.* UCP2 transports C4 metabolites out of mitochondria, regulating glucose and glutamine oxidation. *Proc Natl Acad Sci U S A* **111**, 960-965 (2014).
6. Yoneshiro, T. *et al.* BCAA catabolism in brown fat controls energy homeostasis through SLC25A44. *Nature* **572**, 614-619 (2019).
7. Sprenger, H. G. *et al.* Cellular pyrimidine imbalance triggers mitochondrial DNA-dependent innate immunity. *Nat Metab* (2021).
