# Supplementary material for: Combinatorial GxGxE CRISPR screen identifies SLC25A39 in mitochondrial glutathione transport linking iron homeostasis to OXPHOS
Source: Nat Commun. 2022 May 5;13:2483. doi: 10.1038/s41467-022-30126-9 (PMC9072411; doi:10.1038/s41467-022-30126-9)
Supplement: Supplementary file 3 — Description of Additional Supplementary Files [file 41467_2022_30126_MOESM3_ESM.pdf]

Description of Additional Supplementary Files

File Name: Supplementary Data 1
Description: Raw data from combinatorial CRISPR screen

File Name: Supplementary Data 2
Description: Summary of all single KO gene x environment interactions

File Name: Supplementary Data 3
Description: π-score and p-values for all gene x gene interactions in the screen

File Name: Supplementary Data 4
Description: Summary of all gene x gene x environment interaction hits in the screen

File Name: Supplementary Data 5
Description: Expression profiles of SLC25 genes in K562 cells

File Name: Supplementary Data 6
Description: Metabolite profiles of SLC25A39 KO in K562 mitochondria

File Name: Supplementary Data 7
Description: Metabolite profiles of SLC25A39 KO in HeLa mitochondria

File Name: Supplementary Data 8
Description: Metabolite profiles of SLC25A39 KO in K562 cells
