# Supplementary material for: Combinatorial GxGxE CRISPR screen identifies SLC25A39 in mitochondrial glutathione transport linking iron homeostasis to OXPHOS
Source: Nat Commun. 2022 May 5;13:2483. doi: 10.1038/s41467-022-30126-9 (PMC9072411; doi:10.1038/s41467-022-30126-9)
Supplement: Supplementary file 12 — Reporting Summary [file 41467_2022_30126_MOESM12_ESM.pdf]

nature research

Corresponding author(s): Hongying Shen
Last updated by author(s): 04/10/2022

Reporting Summary

Nature Research wishes to improve the reproducibility of the work that we publish. This form provides structure for consistency and transparency in reporting. For further information on Nature Research policies, see our Editorial Policies and the Editorial Policy Checklist.

Statistics

For all statistical analyses, confirm that the following items are present in the figure legend, table legend, main text, or Methods section.

n/a	Confirmed	
☐	☒	The exact sample size (n) for each experimental group/condition, given as a discrete number and unit of measurement
☐	☒	A statement on whether measurements were taken from distinct samples or whether the same sample was measured repeatedly
☐	☒	The statistical test(s) used AND whether they are one- or two-sided<br>*Only common tests should be described solely by name; describe more complex techniques in the Methods section.*
☐	☒	A description of all covariates tested
☐	☒	A description of any assumptions or corrections, such as tests of normality and adjustment for multiple comparisons
☐	☒	A full description of the statistical parameters including central tendency (e.g. means) or other basic estimates (e.g. regression coefficient) AND variation (e.g. standard deviation) or associated estimates of uncertainty (e.g. confidence intervals)
☐	☒	For null hypothesis testing, the test statistic (e.g. F, t, r) with confidence intervals, effect sizes, degrees of freedom and P value noted<br>*Give P values as exact values whenever suitable.*
☒	☐	For Bayesian analysis, information on the choice of priors and Markov chain Monte Carlo settings
☒	☐	For hierarchical and complex designs, identification of the appropriate level for tests and full reporting of outcomes
☐	☒	Estimates of effect sizes (e.g. Cohen's d, Pearson's r), indicating how they were calculated

Our web collection on statistics for biologists contains articles on many of the points above.

Software and code

Policy information about availability of computer code

Data collection	Xcalibur 4.1 (Thermo Scientific) for mass spec data acquisition and Compound discoverer 3.1 (Thermo Scientific) for mass spec data processing. RT-qPCR data was collected using StepOne Software (version 2.1 Applied Biosystems). Western blot data was imaged/collected using image Lab Touch Software in ChemiDoc MP (version 2.4.0.03 BioRad).
Data analysis	Xcalibur 4.1 (Thermo Scientific) and Tracefinder 4.1. (Thermo Scientific) for mass spec data analysis. GraphPad 7.0a (Prism) was used for statistics for cell characterization and mass spec data. R version 3.6.0 and Bioconductor (r3.9) were used for screen data analysis. RT-qPCR data was analyzed using StepOne Software (version 2.1 Applied Biosystems).

For manuscripts utilizing custom algorithms or software that are central to the research but not yet described in published literature, software must be made available to editors and reviewers. We strongly encourage code deposition in a community repository (e.g. GitHub). See the Nature Research guidelines for submitting code & software for further information.

Data

Policy information about availability of data

All manuscripts must include a data availability statement. This statement should provide the following information, where applicable:

- Accession codes, unique identifiers, or web links for publicly available datasets
- A list of figures that have associated raw data
- A description of any restrictions on data availability

The screen data are available within the paper and its supplementary data files. Source data are provided with this paper.

Field-specific reporting

Please select the one below that is the best fit for your research. If you are not sure, read the appropriate sections before making your selection.

☒ Life sciences ☐ Behavioural & social sciences ☐ Ecological, evolutionary & environmental sciences

For a reference copy of the document with all sections, see nature.com/documents/nr-reporting-summary-flat.pdf

Life sciences study design

All studies must disclose on these points even when the disclosure is negative.

Sample size	The CRISPR screen was performed in two independent batches. Cell proliferation and mass spec experiments were performed with n = 3 independent biological samples unless stated otherwise. Sample sizes were predetermined based on effect size, standard deviation and significance level required from previous studies or on available literature.
Data exclusions	no exclusion criteria were used for analysis.
Replication	All cell proliferation and mass spec experiments were perform at least two independent times. Replicate experiments were successful.
Randomization	N/A All experiments were done in vitro.
Blinding	N/A All experiments were done in vitro.

Reporting for specific materials, systems and methods

We require information from authors about some types of materials, experimental systems and methods used in many studies. Here, indicate whether each material, system or method listed is relevant to your study. If you are not sure if a list item applies to your research, read the appropriate section before selecting a response.

Materials & experimental systems

n/a	Involved in the study	
☐	☒	Antibodies
☐	☒	Eukaryotic cell lines
☒	☐	Palaeontology and archaeology
☒	☐	Animals and other organisms
☒	☐	Human research participants
☒	☐	Clinical data
☒	☐	Dual use research of concern

Methods

n/a	Involved in the study	
☒	☐	ChIP-seq
☒	☐	Flow cytometry
☒	☐	MRI-based neuroimaging

Antibodies

Antibodies used	Anti-SLC25A39 (Proteintech, 14963-1-AP, 1:100); Anti-VDAC (Cell Signaling Technology, 4661T, 1:1000); Anti-SHMT2 (Sigma-Aldrich, HPA-020549, 1:1000); Anti-Calreticulin (Cell Signaling Technology, 12238T, 1:1000); Anti-LAMP2 (Santa Cruz Biotechnology, sc-18822, 1:1000); Anti-NDUFA7 (Proteintech, 15300-1-AP, 1:500); total OXPHOS human WB antibody Cocktail (Abcam, ab110411, 1:1000); GAPDH (Invitrogen, 39-8600, 1:1000).
Validation	validation statements have been provided on the company website.<br>Anti-SLC25A39: validated using the CRISPR KO cells and overexpression cells. https://www.ptglab.com/products/SLC25A39-Antibody-14963-1-AP.htm<br>Anti-VDAC: https://www.cellsignal.com/products/primary-antibodies/vdac-d73d12-rabbit-mab/4661<br>Anti-SHMT2: https://www.sigmaaldrich.com/US/en/product/sigma/hpa020549<br>Anti-Calreticulin: https://www.cellsignal.com/products/primary-antibodies/calreticulin-d3e6-xp-rabbit-mab/12238<br>Anti-LAMP2: https://www.scbt.com/p/lamp-2-antibody-h4b4<br>Anti-NDUFA7: https://www.ptglab.com/products/NDUFA7-Antibody-15300-1-AP.htm<br>total OXPHOS human WB antibody Cocktail: https://www.abcam.com/total-oxphos-human-wb-antibody-cocktail-ab110411.html<br>GAPDH: https://www.thermofisher.com/antibody/product/GAPDH-Antibody-clone-ZG003-Monoclonal/39-8600

Eukaryotic cell lines

Policy information about cell lines

Cell line source(s)	K562 (ATCC CCL-243), HeLa Cells (cloned in the De Camilli lab, ATCC), HEK293T(ATCC CRL-3216),HEK293FT(Thermo Fisher R70007)
Authentication	STR profiling
Mycoplasma contamination	Cell lines were verified to be free of mycoplasma contamination
Commonly misidentified lines (See ICLAC register)	The cell lines used in the study were obtained from ATCC and Thermo Fisher.
